# Traditional chinese medicine syndromes classification associates with tumor cell and microenvironment heterogeneity in colorectal cancer: a single cell RNA sequencing analysis

**DOI:** 10.1186/s13020-021-00547-7

**Published:** 2021-12-07

**Authors:** Yiyu Lu, Chungen Zhou, Meidong Zhu, Zhiliang Fu, Yong Shi, Min Li, Wenhai Wang, Shibo Zhu, Bin Jiang, Yunquan Luo, Shibing Su

**Affiliations:** 1 Institute of Interdisciplinary Integrative Medicine Research, Shanghai University of Traditional Chinese Medicine, Shanghai, 201203 China; 2 Department of Anorectal, Nanjing Hospital of Chinese Medicine, Nanjing University of Chinese Medicine, Nanjing, 210001 China; 3 Department of Liver and Gallbladder Surgery, Shuguang Hospital, Shanghai University of Traditional Chinese Medicine, Shanghai, 201203 China; 4 Cinoasia Institute, Shanghai, 200438 China; 5 Department of Oncology, Nanjing Hospital of Chinese Medicine, Nanjing University of Chinese Medicine, Nanjing, 210001 China; 6 Department of Oncology, Shanghai Baoshan Hospital of Integrated Traditional Chinese Medicine and Western Medicine, Shanghai University of Traditional Chinese Medicine, Shanghai, 201999 China; 7 Center for Pharmacogenomics, School of Life Sciences, Fudan University, Shanghai, 200438 China

**Keywords:** Excess and Deficiency **s**yndromes classification, Colorectal cancer, Tumor heterogeneity, Tumor microcirculation, Single cell RNA sequencing

## Abstract

**Background:**

Colorectal cancer (CRC) is one of the common gastrointestinal malignancies, tumor heterogeneity is the main cause of refractory CRC. Syndrome differentiation is the premise of individualized treatment of traditional Chinese medicine (TCM), but TCM syndrome lacks objective identification in CRC. This study is to investigate the correlation and significance of tumor heterogeneity and TCM syndromes classification in CRC.

**Methods:**

In this study, we using scRNA-seq technology, investigate the significance of tumor heterogeneity in TCM syndromes classification on CRC.

**Results:**

The results showed that 662 cells isolated from 11 primary CRC tumors are divided into 14 different cell clusters, and each cell subtype and its genes have different functions and signal transduction pathways, indicating significant heterogeneity. CRC tumor cell clusters have different proportions in Excess, Deficiency and Deficiency-Excess syndromes, and have their own characteristic genes, gene co-expression networks, gene functional interpretations as well as monocle functional evolution. Moreover, there were significant differences between the high expressions of MUC2, REG4, COL1A2, POSTN, SDPR, GPX1, ELF3, KRT8, KRT18, KRT19, FN1, SERPINE1, TCF4 and ZEB1 genes in Excess and Deficiency syndrome classification in CRC (*P* < 0.01).

**Conclusions:**

The Excess and Deficiency syndromes classification may be related to tumor heterogeneity and its microenvironment in CRC.

## Background

Colorectal cancer (CRC) is one of the most common digestive tract cancers. It ranks third in the most common malignant tumors in the world. The incidence rate increased from 12 million cases in the early 70 s to 56 million cases now, with an annual growth rate of 4.2%, and mortality rate ranks second of the malignant tumor death spectrum [1, 2], seriously endangers human health. In addition to genetic and living habits, the heterogeneity of tumor and microenvironment may be the main internal cause in CRC [3, 4], it may be related to different cell subpopulations, gene status and phenotypes in tumors [5, 6].

High throughput cellular RNA sequencing technology has been widely used in transcriptome analysis to study transcriptional structure, splicing patterns, gene and transcriptional expression levels [7]. However, this sequencing method cannot be specific to a single cell, blurring the characteristics of different cell groups. Recently developed single cell RNA sequencing (scRNA-seq) technology is used to measure gene expression at the single cell level in cancer research [8]. It provides higher cell differential resolution than high-throughput RNA sequencing, and can analyze all cell types, gene expression profiles, characteristic genes and biological function evolution of all tumor cell types [9]. It has been applied to the study of tumor heterogeneity in some cancers, such as lung cancer [10], breast cancer [11], liver cancer [12] and CRC [13, 14].

Tumor heterogeneity is one of the characteristics of malignant tumors. In the process of tumor growth, after multiple divisions and proliferation of cells, its daughter cells show changes in molecular biology or genes, resulting in differences in tumor growth rate, invasion ability, sensitivity to drugs, prognosis, etc. [5, 6]. With the development of single-cell sequencing technology, it has been increasingly used for CRC tumor heterogeneity research. Recent researches have used single-cell RNA sequencing [14–20], single-cell DNA sequencing [21] and single-cell multi-omics [13, 22] technologies, from tumor cells [13–16], stem cells [17], circulating tumor cells (CTC) [18], granulocytes[13], T and B lymphocytes [19, 20], etc., explored their genetic heterogeneity, phenotypic heterogeneity, cell signaling pathway heterogeneity and single cell lineage development heterogeneity of tumor cells and their microenvironment in CRC. It improves our understanding of tumor heterogeneity in CRC, and may provide important information for clinical prognosis, identification of diagnostic markers, and personalized cancer treatment.

Treatment based on syndromes differentiation is a common method in the treatment of CRC in traditional Chinese medicine (TCM), the syndromes differentiation is the premise of individualized treatment in TCM, but the TCM syndrome, also called Zheng, is usually identified by the clinical manifestations of patients by the observation and the empirical judgment of TCM physicians, lack of objective detection and diagnosis [23]. Using single-cell sequencing technology to carry out biological detection can provide an objective data for TCM syndrome differentiation. This study used the second-generation scRNA-seq technology, Smart-seq2 to analyze the subgroup classification, characteristic genes and subgroup gene co-expression network, gene ontology (GO) and The Kyoto Encyclopedia of Genes and Genomes (KEGG) signal transduction pathways and changes in single-cell lineage development, explored the significance of tumor cells and their microcirculation heterogeneity in the TCM syndromes classification of CRC.

## Materials and methods

### Patients

In this study, 11 patients (7 males and 4 females) were enrolled at Shuguang hospital, Shanghai University of Traditional Chinese Medicine and Nanjing hospital of Traditional Chinese Medicine, Nanjing University of Traditional Chinese Medicine from March 2019 to February 2020. Among, the age ranged from 43 to 72 years, with a median of 64 years (Table 1). The diagnostic criteria of CRC follow the Diagnosis and Treatment Regulations on CRC (2010 Edition) issued by the Ministry of Health of the People’s Republic of China [24]. The TCM syndrome differentiation for CRC were according to the Guiding Principles for Clinical Research on New Drugs in TCM (3rd edition) [25] and TCM diagnostics (tenth edition). Every case was pattern identified by three TCM oncologists in associated chief position, and the collected data were assessed by Chi-square test for their consistency, and final judgment was made by a chief TCM oncologist. This research project was approved with the local ethics committee of Shuanghai University of TCM, and all patients were informed consent for this study.

**Table 1:** Clinical characteristics and TCM syndrome types of CRC patients

Patients (PT)	Age	Sex	Histopathological diagnosis	Stages	TNM	Tumor sizes (CM)	TCM syndromes
PT1	69	F	Rectal mushroom type	III b	T3N1bM0	4.0 × 3.5	DES
PT2	43	M	Transverse colon mushroom/ulcer type	II a	T3N0M0	4.7 × 3.5	ES
PT3	49	F	Ascending colon mushroom/ulcer type	II a	T3N0M0	9.0 × 8.5 × 4.0	DS
PT4	58	F	Rectal and sigmoid colon junction ulcer type	II a	T3N0M0	6.0 × 4.0	ES
PT5	72	F	Rectal ulcer type	III b	T3N2bM0	3.5 × 2.0	DES
PT6	60	M	Rectal ulcer type	II a	T3N0M0	7.5 × 6.0 × 0.8	DS
PT7	64	M	Rectal ulcer type	III b	T3N1M0	3.0 × 3.0	ES
PT8	64	M	Sigmoid colon mushroom/ulcer type	III b	T3N1bM0	6.5 × 4.0	ES
PT9	68	M	Rectal and sigmoid colon junction ulcer type	II a	T3N0M0	2.0 × 3.0	ES
PT10	67	M	Rectal ulcer type	II a	T3N0M0	2.5 × 2.5	DS
PT11	51	M	Rectal ulcer type	II a	T3N0M0	2.5 × 2.5 × 2.0	DES

### Sample collection and preparation

The collected fresh tumor was from the above patients after surgical resection. Tumor tissue (TT) were transferred into pre-warmed DMEM medium containing 2 mg/ml collagenase P (Roche, USA) and 10 U/µl DNase I (Roche, USA), digested at 37 ℃, for 60 min, filter and centrifuge at 400 × g for 5 min, and then employed fluorescence activated cell sorting (FACS) to select live cells, After staining with 3 nM CFSE for 5 min, live single cells were manually selected.

### ScRNA-seq library preparation and sequencing

ScRNA-seq was performed according to the manufacturer’s instructions of Smart-seq 2 (12). After lysis of single cells, reverse transcription was performed using Superscript reverse transcriptase (Takara, Japan) and locked TSO oligonucleotides, (Exiqon, Denmark). Full-length cDNA preamplification was conducted with 22 cycles of PCR amplification and HiFi-HotStart ReadyMix (KAPA Biosystems, USA). Subsequently, Ampure XP beads (Beckman, Brea, CA, USA.) were used for PCR purification. An Agilent high-sensitivity DNA chip was used to ensure the size and distribution of the cDNA library. Barcoded libraries were fragmented and tagged using a Nextera XT DNA sample preparation kit (Illumina). Then, we used reagents from the Nextera XT kit to amplify adapter-ligated fragments. Pooled libraries with unique N5-N7 barcodes were sequenced using a HiSeq 2500 instrument (Illumina) and single-end 50-bp read flow cells.

### ScRNA-seq data pre-processing and quality control

Fastq reads were initially filtered using Trimmomatic. The remaining clean reads were aligned to UCSC human genome 19 (hg19) using Hisat 2. Next, we used Feature Counts software to quantify the expression of each gene, and counts were obtained for each sample. The expression level of each gene was converted to a transcript per million (TPM) value. Then, the expression values were log-normalized.

### ScRNA-seq analysis

The ScRNA-seq analysis workflow included t-distribution stochastic neighbor embedding (tSNE) projection of each single cell, cluster analysis, identification of differentially expressed genes (DEGs) for each cell subpopulation, gene co-expression networks analysis, pathway enrichment analysis, and single cell trajectory analyses.

### Unsupervised clustering and DEGs analyses

The expression tables of all cells were fed into an iteratively unsupervised clustering pipeline. We determined that the cells in the same cluster acted as the same subtype based on key genes mapping of different cell types using ‘Seurat’ package (V3.1.2). To assign gene markers for single cell clusters, DEGs were identified by calculating fold-change and p values between different groups using t-test method. We set a 1.5-fold cutoff of fold change and a false discovery rate (FDR) to *P* < 0.05, as the criteria for DEGs selection. This was determined using the ‘stats’ in R. DEGs heatmaps were generated with ‘Heatmap’ package (V1.0.12).

### Gene co-expression network and pathway enrichment analyses

To construct the gene co-network, we constructed the network adjacency between genes, i and j, according to Pearson’s correlation between their expression profiles among cells. We then obtained the gene-to-gene co-expression adjacency matrix by computing the correlation co-efficient for these genes. Next, we selected the genes with high correlations (0.8 or greater) to draw a co-expression network graph using Cytoscape version 3.6.1. The pathway enrichment analysis was based on Gene Ontology Biological Processes (GOBP) andKEGG profiling by Metascape (http://metascape.org/) using *P* Value Cutoff 0.01. We selected a subgroup of representative genes from each cluster compared to the rest clusters for pathway enrichment and functional annotation.

### Single cell trajectory analysis

To characterize the potential process of CRC cell functional changes and determine the potential lineage differentiation between diverse CRC cells, we applied Monocle 2 to perform a pseudo-time analysis of the evolution of CRC cells. Cells were chosen based on Seurat cluster identification results. All analyses were performed in R.

### Statistical analysis

All data were expressed as means ± standard error of the mean, and the statistical analysis was performed using GraphPad Prism v8.0. Comparisons between groups were performed using a one-way ANOVA or Kruskal–Wallis or Wilcox test. Correlations between different gene expression profiles were examined by Pearson’s correlation analysis. Chi-square test was used to analyze the proportion of cell subpopulations. *P* < 0.05 was considered statistically significant.

## Results

### Clinical characteristics of patients

We generated scRNA-seq data from 662 cells isolated from tumor tissues of 11 patients with primary CRC, of which passed quality control. As shown in Table 1, CRCs were diagnosed pathologically, including 3 cases of colon cancer, 6 cases of rectal cancer, and 2 cases of borderline cancer of the rectum and colon. The clinical characteristics of these participants were recorded at the time of recruitment, including histopathological diagnosis, TNM stage, clinical stage, tumor size, and TCM syndrome type. According to TNM stage (AJCC 8 version), there were 7 cases in stage IIa and 4 cases in stage IIIb. All patients had a histopathological diagnosis of moderately differentiated adenocarcinoma. There were 5 cases in Excess syndrome (ES), 3 cases in Deficiency syndrome (DS), and 3 cases in Deficiency-Excess syndrome (DES).

### Distribution of tumor cell subpopulations in CRC with TCM syndromes

**Fig. 1:**
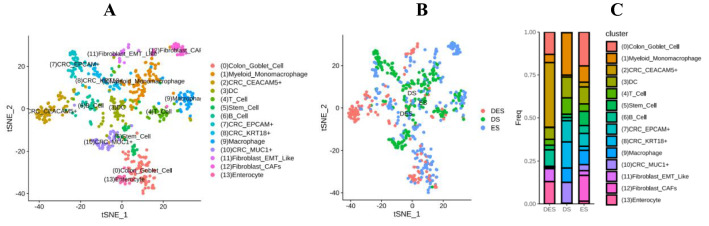
TSNE distribution of cell subpopulation and the proportion in CRC with Excess syndrome (ES), Deficiency syndrome (DS) and Excess-Deficiency syndrome (DES). **A** tSNE distribution of cell subpopulations; **B** the proportion of the three syndromes; **C** columnar graphic of cell subpopulations tSNE distribution and the three syndromes proportion

In order to clarify the relationship between CRC with TCM syndromes and the heterogeneity of intra-tumoral cells and their microenvironment, we analyzed the distribution of tumor single cell subpopulations of the three types of ES, DS and ESDS in CRC. The clustering analysis identified to partition the cells into 14 clusters. These clusters could be assigned to nine known cell lineages through marker genes, including marked with carcinoembryonic antigen-related cell adhesion molecules 5 (CEACAM5), epithelial cell adhesion molecules (EPCAM) ^+^, Keratin 18 (KRT18) ^+^ and transmembrane glycoprotein Mucin 1 (MUC1) ^+^ 4 types of CRC cells, colon goblet cell, myeloid-derived monomacrophage, dendritic cells (DC), T lymphocytes, stem cells, B lymphocytes, macrophage, epithelial-mesenchymal transition (EMT) -like fibroblasts, EMT, cancer-related fibroblasts (CAFs) and enterocyte. The proportion of each cell lineage varies greatly among different samples (Fig. 1A and C), indicating there were the heterogeneity of single cells and their microenvironment in the tumor of CRC.

Moreover, we found that the percentages of cell subpopulations of CRC with ES, DS and DES were different (Table 2). The main distribution of cells in DES is CEACAM 5^+^ cells (37.67%). The main distribution in DS were myeloid-derived monomacrophages (24.71%), KRT18^+^ cells (15.21%), DC (12.17%) and MUC1^+^ cells (12.17%). Goblet cells (19.76%) and CAFs (15.02%) were the most distributed in ES (Fig. 1B and C).

**Table 2:** Distribution of cell subpopulations in TCM syndrome types of CRC

Cell types	Cell subpopulations	DES/Numbers (%)	DS/Numbers (%)	ES/Numbers (%)	P*
CRC cell	CEACAM5 +	55 (37.67)	3 (1.14)	7 (19.76)	
	EPCAM +	0 (0.00)	32 (12.17)	19 (7.51)	
	KRT18 +	1 (0.68)	40 (15.21)	6 (2.37)	
	MUC1 +	1 (0.68)	32 (12.17)	9 (3.56)	
	Total	57 (39.04)	107 (40.68)	41 (16.21)	< 0.001
Immune cell	Myeloid monomacrophage	7 (4.79)	65 (24.71)	24 (9.49)	
	DC	10 (6.85)	32 (12.17)	25 (9.88)	
	T cell	5 (3.42)	25 (9.51)	11 (4.35)	
	B cell	14 (9.59)	5 (1.90)	11 (4.35)	
	Macrophage	0 (0.00)	22 (8.37)	21 (8.30)	
	Total	36 (24.66)	149 (56.65)	92 (36.36)	< 0.001
Stem cell	Stem cell	4 (2.74)	5 (1.90)	21 (8.30)	< 0.005
Fibroblast	EMT Like	11 (7.53)	0 (0.00)	7 (2.77)	
	CAFs	0 (0.00)	1 (0.38)	38 (15.02)	
	Total	11 (7.53)	1 (0.38)	45 (17.79)	< 0.001
Enterocyte	Enterocyte	19 (13.01)	0 (0.00)	4 (1.58)	< 0.001
Goblet cell	Goblet cell	19 (13.01)	1 (0.38)	50 (19.76)	< 0.001
Total	14	146	263	253	

^*^Chi-square test

In DES, the proportions of subpopulations of CRC cells, immune cells, stem cells, fibroblasts, colon epithelial cells and goblet cells accounted for 39.04, 24.66, 2.74, 7.53, 13.01, 13.01% respectively. In DS, that were 40.68, 56.65, 1.90, 0.38, 0 and 0.38%, respectively. In ES, the proportions were 16.21, 36.36, 8.30, 17.79, 1.58 and 19.76% respectively. Moreover, there were statistically significant differences (*P* < 0.005) (Table 2). It was indicated that the TCM syndrome classification in CRC may be related to the distributions of intra-tumor cell subpopulations and their heterogeneity.

### Differential gene and functional annotation of tumor cell subpopulations in CRC with TCM syndromes

**Fig. 2:**
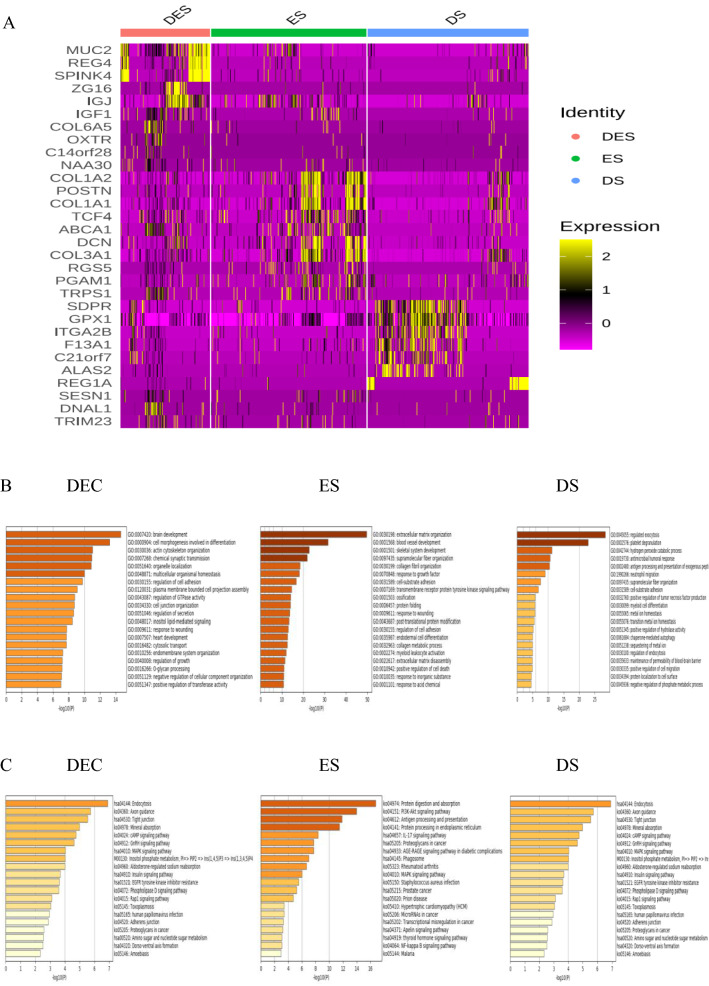
DEGs and functional annotations of tumor cells in CRC with Excess syndrome (ES), Deficiency syndrome (DS) and Excess-Deficiency syndrome (DES). **A** Top30 heat map of DEGs, in the expression level, yellower color was higher and redder color was lower; **B** GO/BP enrichment analysis; **C** KEGG signal pathway enrichment analysis

In order to clarify the relationship between TCM syndromes and cell phenotype in CRC, we analyzed theDEGs and functional annotations of tumor single cell subpopulations in CRC with TCM syndromes. We profiled the top 30 DEGs from distinct single cell subpopulations in CRC with the three types of syndromes using a heatmap (Fig. 2A). There were 686 DEGs (up-regulated 453, down-regulated 233) in ES, 1883 DEGs 1497 (up-regulated 1497, down-regulated 386) in DES and 2,295 DEGs (up-regulated 247, down-regulated 2048) in DS. As shown in Fig. 2, the high expressions of transmembrane glycoprotein Mucin 2 (MUC2) and regenerating islet-derived protein 4 (REG4) in DES were mainly related to brain development, cellogenesis, mitogen-activated protein kinase 1 (MAPK) and cyclic adenosine monophosphate (cAMP) signaling pathways. The high expressions of collagen type I alpha 2 (COL1A2) and periostin (POSTN) genes in ES were mainly related to vascular development and skeletal muscle development, and PI3k-Akt pathway. The high expressions of caveolae-associated protein 2 (CAVIN2 or SDPR) and glutathione peroxidase 1 (GPX1) genes in DS were mainly related to exocytosis, platelet decomposition and endocytosis (Fig. 2B and C), indicating that the TCM syndromes in CRC and may be related to the heterogeneity of genes and their function and signaling pathways in tumor cell subpopulations.

### Co-expression network of high-expressed genes in tumor single cell of CRC with TCM syndromes

**Fig. 3:**
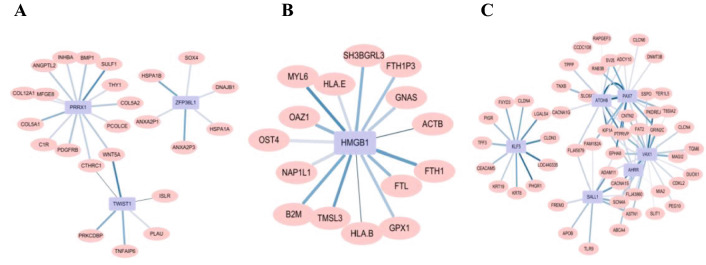
The high-expressed gene co-expression network in CRC with TCM syndromes. **A** Excess syndrome (ES); **B** Deficiency syndrome (DS); **C** Deficiency-Excess syndrome (DES). Pink ovals indicated highly expressed genes, and blue squares indicated TF. The lighter line color indicated lower correlation, and the thicker line indicated smaller p-value

Further, we performed respectively the co-expression network analysis of high-expressed genes in ES, DS and DES with the transcription factor (TF) through spearman correlation, and screened them with a correlation coefficient greater than 0.5 or less than -0.5 and BH less than 0.05. The results were as shown in Fig. 3, the high expressed genes in the DES were mainly protein atonal homolog 8 (ATOH8), paired box protein Pax 7 (PAX7), ventral anterior homeobox 1 (VAX1), and their functions were mainly related to the reaction of purine-containing compounds, the secretion and decomposition of cortisol. The highly expressed gene in the DS was high mobility group protein B1 (HMGB1), and it related to iron ion transport and growth and development functions. The highly expressed genes in the ES were paired mesoderm homeobox protein 1 (PRRX1), mRNA decay activator protein ZFP36L (ZFP36L1) and twist-related protein 1 (TWIST1), and their functions were importantly related to embryogenesis and angiogenesis.

### Correlation of TCM syndromes and EMT-related DEGs of tumor single cells in CRC

In order to find potential molecular markers of TCM syndromes in CRC, we conducted Kruskal–Wallis test to screen keratin and EMT-related DEGs of tumor single cells in CRC with TCM syndromes. We found epithelium-specific ETS transcription factor 3 (ELF3), keratin 8, 18 and 19 (KRT8, 18 and 19), fibronectin 1 (FN1), serpin family Emember 1 (SERPINE1), transcription factor 4 (TCF4) and zinc finger E-box binding homeobox 1 (ZEB1) genes have significant differences in ES, DS and DES of CRC (*P* < 0.01) (Table 3).

**Table 3:** Screening of EMT-related DEGs in tumor single cells in CRC

Genes	P^*^	BH
ELF3	1.12E−07	6.32E−07
KRT7	0.105616809	0.179548575
KRT8	1.36E−08	1.15E−07
KRT18	0.003262755	0.009244472
KRT19	2.13E−10	3.63E−09
CDH1	0.122199768	0.188854187
GRHL1	0.247310338	0.28028505
FN1	1.46E−05	6.19E−05
SERPINE1	0.046275357	0.098335134
TCF4	2.06E−05	7.01E−05
VIM	0.208640718	0.253349443
TGFB2	0.173304473	0.226628926
TGFB3	0.164412474	0.226628926
SIX1	0.782746466	0.789963446
NOTCH2	0.789963446	0.789963446
ZEB1	0.006163609	0.014968766
ZEB2	0.060271568	0.113846294

^*^Kruskal–Wallis test

**Fig. 4:**
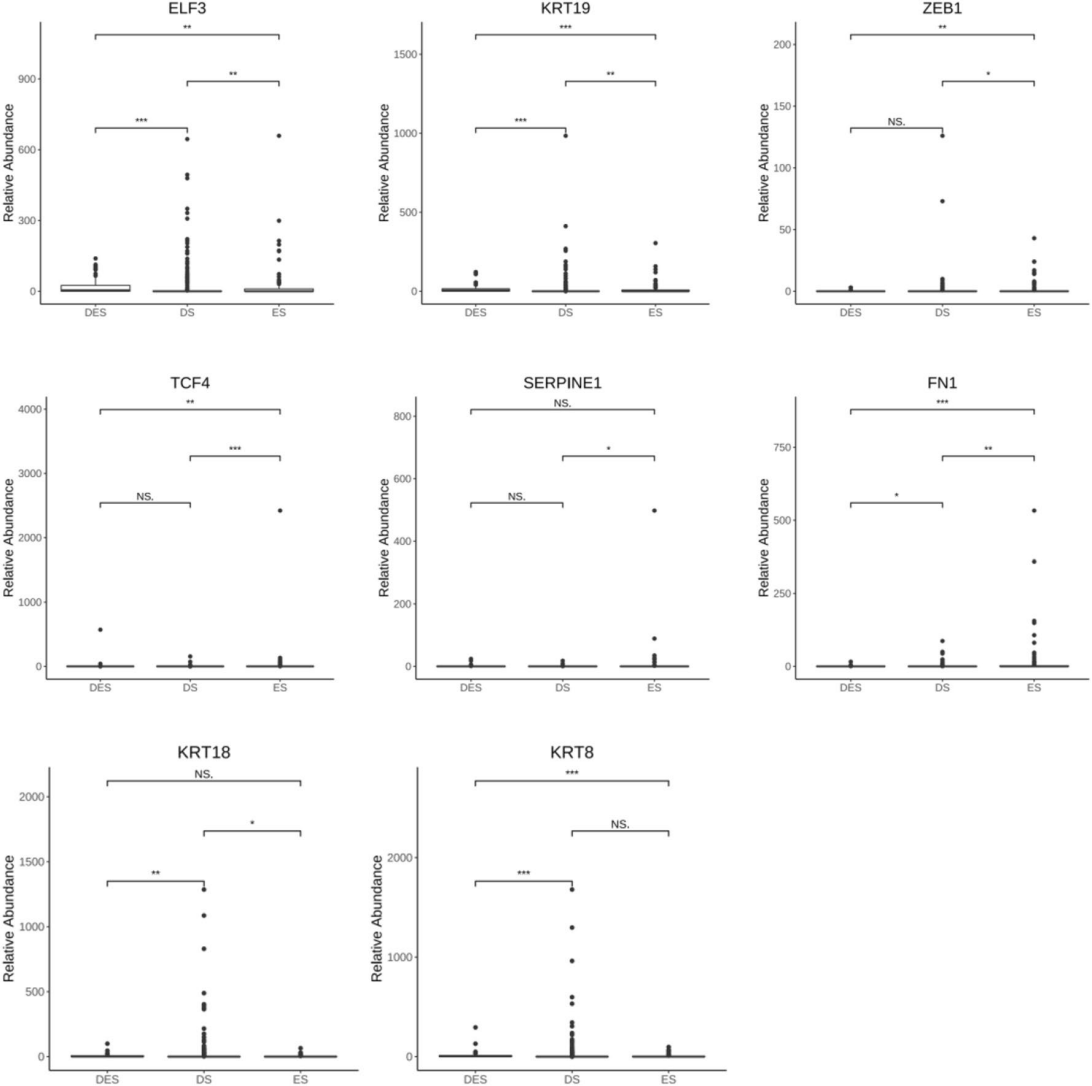
Correlation of ELF3, KRT8, KRT18, KRT19, FN1, SERPINE1, TCF4 and ZEB1 with Excess syndrome (ES), Deficiency syndrome (DS) and Excess-Deficiency syndrome (DES) in CRC. Analysis of variance, Wilcox test, *P < 0.05; **P < 0.01; ***P < 0.001; NS, no statistical significance

Furthermore, we used the Wilcox test to compare the roles of ELF3, KRT8, KRT18, KRT19, FN1, SERPINE1, TCF4 and ZEB1 genes in TCM syndrome differentiation of CRC with ES, DS and DES. The results were as shown in Fig. 4, ELF3, KRT18, KRT19 and FN1genes have significant difference between DES and DS or ES, ES and DS (*P* < 0.05 or *P* < 0.01 or *P* < 0.001); TCF4 and ZEB1 genes have significant difference between DES and ES or ES and DS (*P* < 0.05 or *P* < 0.01 or *P* < 0.001); KRT8 gene has significant differences between DES and ES or DS (*P* < 0.001); SERPINE1 gene has significant difference between ES and DS (*P* < 0.05), but there was no statistical difference between other TCM syndrome types (*P* > 0.05).

### Differences in the evolution of tumor cell subpopulations in TCM syndromes of CRC

**Fig. 5:**
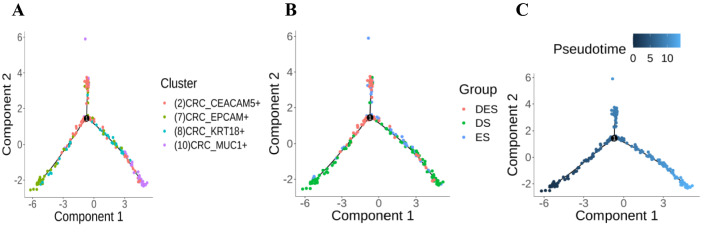
Monocle pseudotime analysis of tumor cell subpopulation evolution in CRC with TCM syndromes. **A** The evolutionary trajectory of CEACAM5 + , EPCAM + , KRT18 + and MUC1 + tumor cell subpopulations; **B** the distribution of Evidence (ES), Deficiency Syndrome (DS), and Deficiency and Excessive Syndrome (DES) on the evolutionary trajectory of tumor cell subpopulations; **C** the pseudotime trajectory of tumor cell subgroup evolution. The darker color is 'earlier', the pseudotime is 0; the lighter color is the more ‘late’, the pseudotime is greater

In order to clarify the relationship between TCM syndromes and the function evolution among tumor cell subpopulations in CRC, we used monocle (V2.16.0) to construct the cell chronological trajectory of the 4 subpopulations of tumor cells. The results were as shown in Fig. 5, the evolution trajectory of the subpopulation starts from the lower left corner to the lower right corner, from EPCAM^+^ cell subgroup, through CEACCAM5^+^ cell subgroup and KRT18^+^ cell subgroup, evolving MUC1^+^ cell subgroup. DES was mainly in the middle of the trajectory, ES was in the second half of the trajectory, and DS was all over the entire trajectory.

## Discussion

CRC has been called “Intestinal accumulation”, “Accumulation” or “Intestinal mushroom” in TCM. Modern Chinese medicine physicians considered that the occurrence of CRC is due to the deficiency of Vital Qi, and the stagnation and accumulation of Evil Qi such as “Damp heat”, “Blood stasis” and “Poison stagnation” in the large intestine for long time, which resulted in the main pathogenesis in TCM of CRC is the “Deficiency of Vital Qi and Excess of Evil Qi” and “Combination of Deficiency and Excess” [26, 27]. Previous study on the distribution of clinical TCM syndromes in 760 cases of CRC has showed 565 in DS, 81 in ES, 52 in DES and 62 in no syndrome (NS), among DS is the most common syndrome type in CRC patients [27]. In this study, we used Smart-seq2 technology to explore the relationship between the heterogeneity of tumor cells and microcirculation and TCM syndromes in CRC through analyzing the classification of tumor cells and the proportion of each subgroup, the characteristic genes, gene co-expression network, the functional interpretation and the evolution of monocle functions.

Tumor heterogeneity is mainly divided into two types including inter-tumor heterogeneity and intra-tumor heterogeneity [28]. Almendro et al. [29] have classified intra-tumoral heterogeneity into cell, gene and functional heterogeneity, and temporal and spatial heterogeneity under the perspective of systems biology. Aaron S et al. [30] divided it into genetic and, phenotypic heterogeneity, cell signaling and pathway activity heterogeneity, and temporal and spatial heterogeneity. The manifestations of the tumor heterogeneity are complex and changeable. Different forms of heterogeneity may occur throughout the process of tumor formation and development. It needs to pay attention to is the relationship between the changes in molecular mechanisms and different functions in the process of the tumor heterogeneity. In this study, we found that the intra-tumor cells in CRC have 14 different cell subpopulations, 11 known cell lineages, and their genes have different functions and signal transduction pathways, as well as the development of different single cell lineages, showing obvious cells and genes and their phenotypic heterogeneity and cell signaling and pathway activity heterogeneity. Moreover, there were the different distributions of intra-tumor cell subpopulations in different TCM syndromes including ES, DS and DES, and the high expressions of MUC2 and REG4 in DES were mainly related to brain development, cytogenesis, MAPK and cAMP signaling pathways; the high expressions of COL1A2 and POSTN genes in ES were mainly related to vascular development, skeletal muscle development, and PI3k-Akt Pathway; SDPR and GPX1 genes were highly expressed in DS, mainly related to vomiting, platelet decomposition, and endocytosis. These results suggested the correlation of TCM syndromes classification and the heterogeneity of tumor cell subpopulations, and their characteristic genes, phenotypes and signaling pathways in CRC.

The tumor microenvironment includes all the components of non-cancerous solid tumors. It is a complex ecosystem composed of multiple cell types, which play different roles in tumor development, and is highly heterogeneous [31]. This study found 14 different cell subpopulations and 11 known cell lineages among 662 cells isolated from 11 primary CRC tumor tissues, including CRC cancer cells (CEACAM5 + , EPCAM + , KRT 18^+^ and MUC1^+^ cells), stem cells, immune cells (myeloid-monomacrophages, macrophages, DC, Goblet cells, T and B cells), fibroblast cells (ETM like and CAFs) and enterocyte. However, the proportion of cell subpopulations among ES, DS and DES in CRC is different. The main distribution of CEACAM5^+^ cells subpopulation was in DES; myeloid-monomacrophages, KRT 18^+^ cells, DC and MUC1^+^ cells subpopulations were the most distributed in DS; and goblet cells and fibroblasts subpopulations were the most distributed in ES. It was suggested that the TCM syndromes classification may be related to tumor cell subpopulations and the heterogeneity of tumor microenvironment in CRC.

Moreover, the pseudo-time analysis of monocle functional evolution showed that, 4-cell subpopulations of cancer cells in CRC with TCM syndromes appeared in different states and at different pseudo-times. Among, that of DES was mainly distributed in the middle part, ES was in the second part, and DS was all over the trajectory of pseudo-times, indicating that the DS run through the whole process of CRC tumor cell function evolution, the middle stage is the DES, and the later stage is mainly the ES. These results might provide scientific evidence to clarify the main pathogenesis of CRC in TCM, the "Deficiency of Vital Qi and Excess of Evil Qi" and "Combination of Deficiency and Excess" from the approach of tumor cell development.

CAFs are a kind of activated fibroblasts and are important stromal cells in the tumor microenvironment. In CRC, they interact with tumor cells to promote tumor occurrence, development and metastasis [32]. This study identified two cell subpopulations of fibroblasts including CAFs and EMT-like fibroblasts from CRC tumor tissues, which is consistent with the results of Li H et al. [16] in the previous research of tumor single cell in CRC. In this study, an EMT-related gene, fibronectin FN1 was highly expressed, and there was a significant difference between DES and ES, DES and DS or ES and DS in CRC. ZEB1 is a EMT transcription factor, it has been reported that the high expression of ZEB1 correlates with liver metastasis and poor prognosis in CRC [33, 34]. Moreover, TCF4, a transcription factor, participated in the regulation of Wnt/β-catenin signaling pathway in CRC cells [35, 36], and promotes adriamycin resistance and cell stemness by regulating the expression of EMT-related ZEB1 and ZEB2 [37]. This study found that the high expressions of TCF4 and ZEB1 genes have significant difference between DES and ES or ES and DS in CRC. It suggested that CAFs and EMT-related genes, the high expressions of FN1, ZEB1 and TCF4 in the tumor microenvironment may be involved in TCM syndromes classification in CRC.

Cytokeratin is the intermediate filament of the cell body, which can be divided into 20 different types according to its molecular weight and isoelectric point [38]. Among, KRT8, 18 and 19 have been reported to correlate with CRC [39], and their expression changes associate with progression towards neoplasia [40]. Moreover, KRT8 has been indicated epithelial to mesenchymal transition [41], and loss of K8 phosphorylation was also suggested to promote tumor migration and formation of metastasis [42]. KRT 18 and 19 increase in peripheral blood as soluble fragments in CRC [39]. In this study, Smart-seq2 analysis shows that, KRT8, 18 and 19 genes were enriched in the tumor tissues of CRC, and the high expressions of KRT18 and KRT19 genes were significant differences in DES and DS or ES, ES and DS classification, and the high expressions of KRT8 gene has significant differences between DES and ES or DES and DS in patients with CRC.

ELF3 is defined by their highly conserved ETS DNA binding domain and predominant epithelial-specific expression profile [43]. The ELF3 drives beta-catenin transactivation and associates with poor prognosis in CRC [44, 45]. We found that the high expression of ELF3 gene has a significant difference between DES and ES, DES and DS or ES and DS in CRC. SERPINE1, a clade E member of the serine protease inhibitor gene family and a prominent regulator of the pericellular proteolytic microenvironment [46]. In addition to play important roles in cell adhesion, migration and invasion, it has been reported to induce tumor vascularization and promote cell dissemination and tumor metastasis [47], and involved in the survival and prognosis of CRC [48, 49]. In this study, we found that, the high expressions of SERPINE1 has significant difference between ES and DS in CRC. The above results suggest that the high expressions of KRT19, KRT 18, KRT 8, ELF3 and SERPINE1 genes are potential candidates for identifying TCM syndrome types in CRC, which need to be confirmed by further researches.

The treatment based on TCM syndrome classification is the basic method and characteristic in the TCM treatment of CRC. Once understanding the tumor heterogeneity corresponding to different TCM syndromes, we will be able to utilize objective indicators related tumor heterogeneity to assist the diagnosis of TCM syndromes and treatments in CRC. Our results have shown that the different tumor heterogeneity may correlate with the different TCM syndromes in CRC, such as the distributions of intra-tumor cell subpopulations, genes and their function and signaling pathways, and gene co-expression network, DEGs and evolution of tumor cell subpopulations were also involved in TCM syndrome classification. Moreover, there were significant differences between the high expressions of MUC2, REG4, COL1A2, POSTN, SDPR, GPX1, ELF3, KRT8, KRT18, KRT19, FN1, SERPINE1, TCF4 and ZEB1 genes in Excess and Deficiency syndrome classification in CRC. These findings will be further validated by another approaches to finding a or a panel of potential biomarkers to assist the diagnosis of TCM syndromes, and also it may be helpful to further investigate the mechanism of occurrence and transformation of TCM syndromes, and finding therapeutic targets based on the TCM syndrome classification guiding treatments and/or assess the therapeutic effects and explore the mechanism of therapeutic effects in CRC.

## Conclusions

Through the use of Smart-seq2 technology, this study found that CRC tumor cell subpopulations account for different proportions of ES, DS and DES, and have their characteristic genes, gene co-expression networks and their function and functional evolution. It suggested that the TCM syndrome classification in CRC may be related to the tumor heterogeneity and its microenvironment. In the future, the analyzed results of this study will be further validated, and single-cell multi-omics technology will be used to further explore the role of the tumor heterogeneity on the TCM syndromes classification and transformation in CRC.

## Data Availability

All the data used to support the findings of this study are available from the corresponding author upon reasonable request.

## Abbreviations

TCM: Traditional Chinese Medicine
ES: Excessive syndrome
DS: Deficiency syndrome
DES: Deficiency and Excessive Syndrome
NS: No syndrome
CRC: Colorectal cancer
TT: Tumor tissue
DEGs: Differentially expressed genes
scRNA-seq: Single cell RNA sequencing
CTC: Circulating tumor cells
FACS: Fluorescence activated cell sorting
tSNE: T-distribution stochastic neighbor embedding
GO: Gene ontology
KEGG: Kyoto Encyclopedia of Genes and Genomes
GOBP: Ontology Biological Processes
FDR: False discovery rate
EMT: Epithelial–mesenchymal transition
CAFs: Cancer-related fibroblasts
